# Evaluating the efficacy of an active compression brace on orthostatic cardiovascular responses

**DOI:** 10.1371/journal.pone.0187885

**Published:** 2017-11-22

**Authors:** Hadi Moein, Ramandeep Jhalli, Andrew P. Blaber, Victoria E. Claydon, Carlo Menon

**Affiliations:** 1 Menrva Research Group, Schools of Mechatronic Systems Engineering and Engineering Science, Simon Fraser University, Metro Vancouver, British Columbia, Canada; 2 Department of Biomedical Physiology and Kinesiology, Simon Fraser University, Metro Vancouver, British Columbia, Canada; The University of Tokyo, JAPAN

## Abstract

Orthostatic intolerance, one of the principle causes of syncope, can occur secondary to concomitant venous pooling and enhanced capillary filtration. We aimed to evaluate a prototype portable calf active compression brace (ACB) designed to improve orthostatic haemodynamic control. Fourteen healthy volunteers participated in a randomized, placebo controlled, cross-over, double-blind study. Testing consisted of head-upright tilting and walking on a treadmill conducted on two consecutive days with a pair of ACBs wrapped around both calves. The ACB was actuated on one test day, but not on the other (placebo). Wearability, comfort, and ambulatory use of the ACB were assessed using questionnaires. The average calf pressure exerted by the ACB was 46.3±2.2 mmHg and the actuation pressure was 20.7±1.7 mmHg. When considering the differences between ACB actuation and placebo during tilt after supine rest there were trends for a larger stroke volume (+5.20±2.34%, p = 0.05) and lower heart rate (-5.12±2.41%, p = 0.06) with ACB actuation, with no effect on systolic arterial pressure (+4.86±3.41%, p = 0.18). The decrease in stroke volume after ten minutes of tilting was positively correlated with the height:calf circumference (r = 0.464; p = 0.029; n = 22; both conditions combined). The increase in heart rate after ten minutes of tilting was negatively correlated with the height:calf circumference (r = -0.485; p = 0.022; n = 22; both conditions combined) and was positively correlated with the average calf circumference (r = 0.539; p = 0.009; n = 22; both conditions combined). Participants reported good ACB wearability and comfort during ambulatory use. These data verify that the ACB increased stroke volume during tilting in healthy controls. Active calf compression garments may be a viable option for the management of orthostatic intolerance.

## New & noteworthy

We performed a randomised, placebo controlled trial to evaluate the efficacy of an innovative active compression brace (ACB) to improve orthostatic haemodynamic control in fourteen healthy volunteers. The wearability, comfort, and ambulatory use of the device were also evaluated. Device actuation enhanced stroke volume and reduced orthostatic tachycardia. Participants reported good ACB wearability and comfort ratings during ambulatory use. Active calf compression garments may be a viable option for the management of orthostatic intolerance.

Compression therapy · Active compression brace · Shape memory alloys · Orthostatic intolerance · Syncope.

## Introduction

Syncope, or fainting, refers to a transient loss of consciousness and postural tone that typically occurs when upright and is associated with reduced cerebral blood flow [1,2]. The principal causes of syncope are classified as reflex (neutrally-mediated), cardiac (cardiovascular) and syncope due to orthostatic hypotension (OH) [3]. OH has been defined by consensus as a fall in systolic arterial pressure (SAP) of at least 20 mm Hg and/or diastolic arterial pressure (DAP) of at least 10 mm Hg within 3 minutes of standing [4,5]. Patients suffering from OH may feel dizzy, weak or faint upon standing due to an excessive fall in cardiac output (CO) or inadequate vasoconstrictor mechanisms [4,5]. Approximately 30% of all instances of syncope are due to OH, affecting 5% to 11% of the middle-aged adult population and 15% to 30% of individuals aged 65 years and older [6,7]. In patients with OH, applying external compression to the lower limbs or abdomen might diminish venous pooling and capillary filtration, and so assist venous return and the maintenance of CO [3,8–11].

Static external compression stockings are commonly recommended for the management of syncope. They are prescribed for those suffering from orthostatic intolerance, where the external counter-pressure of the lower limbs or abdomen can reduce venous pooling and capillary filtration, thus increase venous return [9,12]. Garments that compress the abdomen have shown greater efficacy compared to calf compression stockings in the prevention of orthostatic intolerance [8,11,13,14]. However, they are reported to be uncomfortable, hard to put on and remove, and are associated with poor patient compliance [15,16]. For this reason, much of the recent focus on compression garments has been on calf compression. Undoubtedly, pooling and filtration in the legs can be significant, with 500 ml of blood accumulation in the static calf within just 10 minutes of 60° head-upright tilting [17]. However, the efficacy of static calf compression stockings in the management of orthostatic intolerance has been challenged [18]. On the other hand, intermittent pneumatic pressure pumps have shown strong potential for the improvement of orthostatic tolerance. They have been shown to improve arterial flow, increasing venous outflow from the lower limbs, venous blood flow velocity and venous pressure [19–22]. However, they restrict patient mobility and are not portable. Considering the concerns raised regarding the poor efficacy and suitability of current compression garments, we believe there is a need for novel ambulatory compression devices that provide the desired haemodynamic benefit for the patients, are comfortable and wearable, and can produce intermittent pressure.

**Fig 1:**
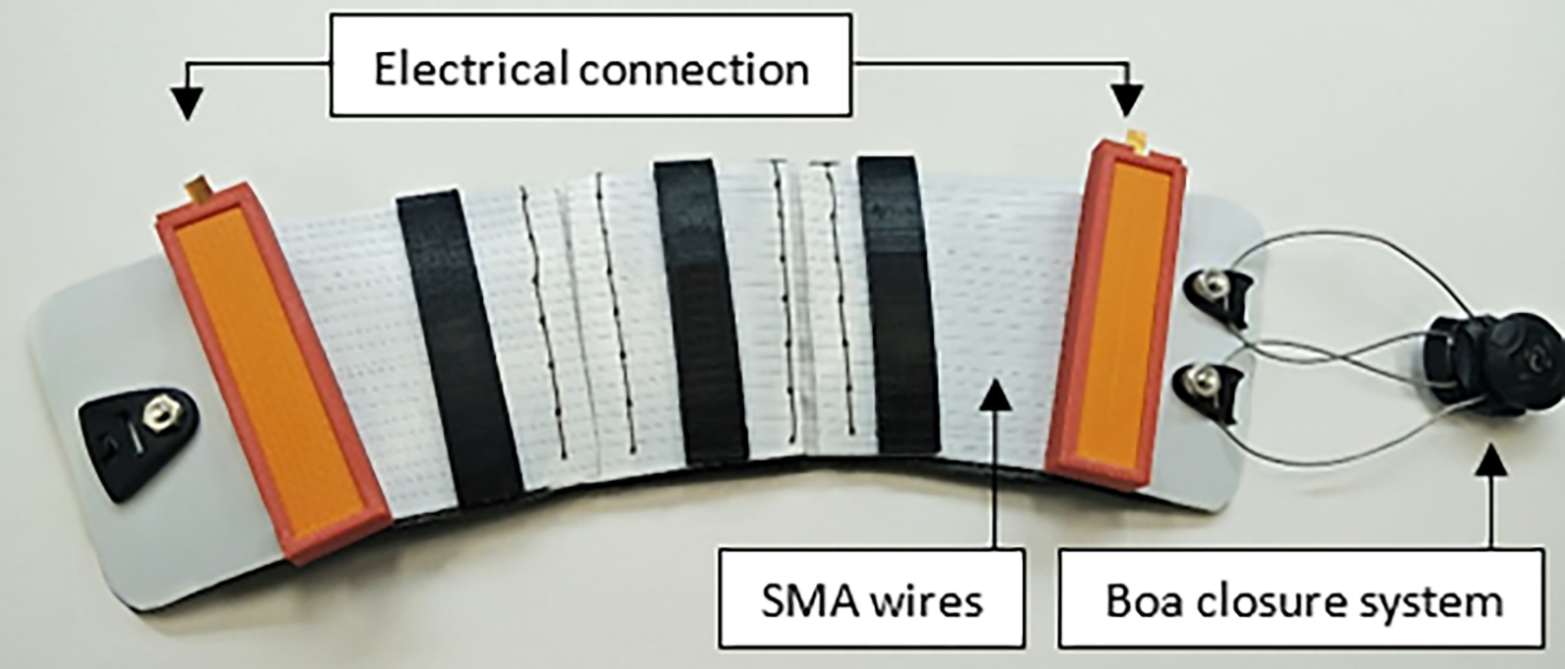
Active compression brace (ACB). The ACB consisted of thin layer of Teflon on top of a protective layer of Neoprene as the body of the brace and SMA wires as the actuators. The 3D printed guides and fixtures with the Boa closure systems, placed at the end of the brace, are also shown in the figure.

We recently developed an active compression brace (ACB) for the calf that utilizes artificial muscle technology. This ACB has been prototyped using smart alloy technology and its performance has previously been investigated in vitro [23]. Specifically, shape memory alloy (SMA) wires were used as actuators in the ACB and produce pressure when actuated. SMAs, including NiTi wires, are a group of metallic alloys that exhibit the characteristics of either large recoverable strains or large force due to temperature and/or load changes. NiTi is one of the most studied SMAs and is commercially available [24,25]. NiTi wires can have high force per unit area (exceeding 200 MPa), and can be actuated using Joule heating via an applied current [25]. The prototyped ACB can be wrapped around the calf area of the leg and applies adjustable initial pressure similar to medical compression stockings using a Boa closure system [26] (Fig 1). The pressure can then be increased by increasing the electrical current applied to the SMA wires.

In this study, we aimed to evaluate the efficacy of the ACB for the improvement of stroke volume (SV) and cardiovascular hemodynamics during orthostatic stress using a randomised placebo-controlled double-blind crossover design. We compared responses with the device activated and with it wrapped but actuation pressure not applied (placebo). We measured the compression on the calves exerted by the ACB and obtained cardiovascular and lower limb circumference changes through beat-to-beat blood pressure monitoring, electrocardiography (ECG), and leg strain gauge plethysmography. We hypothesized that during orthostasis the actuated ACB would reduce the leg volume change, and increase venous return, with an associated increase in SV and decrease in heart rate (HR). We also evaluated the effect of walking on the compression pressures applied, as well as on calf volume changes and cardiovascular responses. Finally, we evaluated the ACB viability, comfort and wearability during ambulation based on participant surveys.

## Methods

### Ethics approval

After the details and procedures of the study had been fully explained to them, all participants gave written informed consent to participate. This study received ethical approval from the Simon Fraser University Office of Research Ethics for all measurements (study #2014s0590). All investigations were performed in association with the Declaration of Helsinki of the World Medical Association.

### Study design

Fourteen adults (four females; aged 27.9±1.3 years) were recruited for this study. Participants completed a brief medical history; all volunteers were healthy and free of cardiovascular and neurological disease. None of the participants were taking any medication. Each participant completed testing on two consecutive days with a pair of ACBs wrapped around both legs. The ACB was not actuated on one testing, and it was actuated on the other testing. The ACBs were placed around the widest part of the calf. The wrapping pressure was adjusted to 15 mmHg in the supine resting position. For the test where we had the ACB actuated, an electrical current of 180 mA per SMA wires was applied to each of the ACBs. Testing was conducted in a randomised double-blind fashion, at the same time of day. Female participants were tested in the same phase of their menstrual cycle by testing on consecutive days. Participants were asked to refrain from strenuous exercise for twelve hours prior to each test, eat a light breakfast, and avoid caffeine on the morning of each test.

Prior to testing, anthropometric measures were taken. Circumference and skinfold thickness of the calf were determined at the widest section of the calf. Calf cross-sectional area (cm^2^) was estimated from the circumference (cm), assuming circularity [27]:

$$Calf\;cross\;sectional\;area={calf\;circumference}^{2}/4\pi$$ (1)

Measurements of circumference and skinfold thickness (cm) were used to estimate adipose tissue cross-sectional area (subcutaneous adipose tissue only):

Adiposetissuecrosssectionalarea=(Calfcrosssectionalarea∙skinfoldthickness)/2 (2)

Muscle cross-sectional areas, also assumed to be circular, were estimated by the difference between cross sectional of areas of the whole limb and adipose tissue with an assumed cross-section of bone with its constituent marrow (6 cm^2^) [27]:

$$Muscle\;cross\;sectional\;area=\frac{{calf\;circumference}^{2}}{4\pi }-\frac{Calf\;cross\;sectional\;area\;∙skinfold\;thickness}{2}-6$$ (3)

### Test protocol

**Fig 2:**
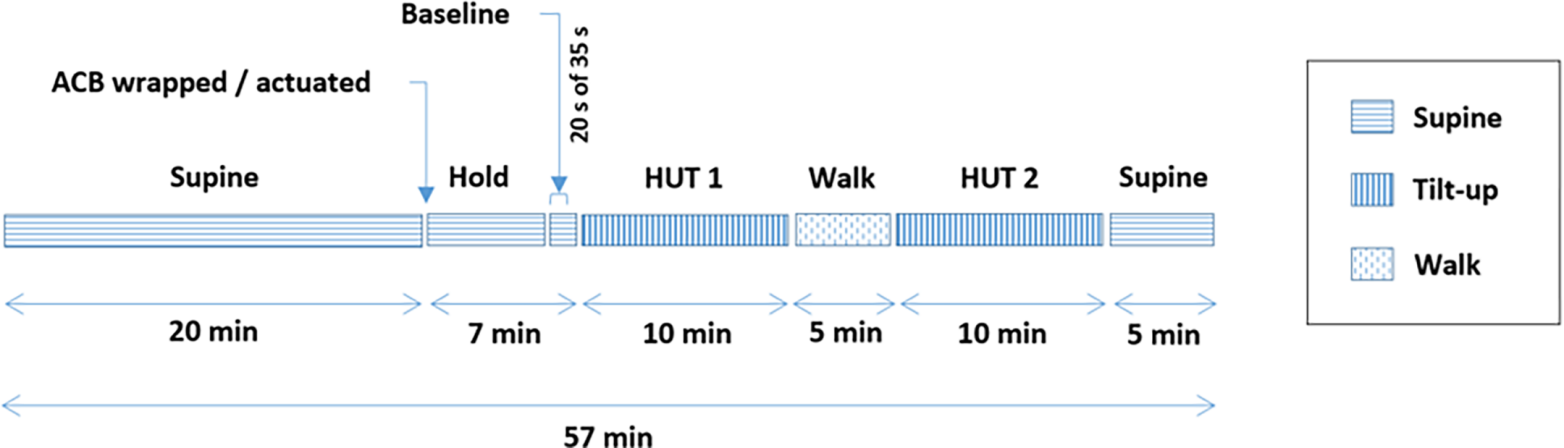
Test protocol. The method of randomisation used in the tests was the true random service [28].

Fig 2 shows the test protocol conducted on each test day. The test consisted of supine rest, head-upright tilting and walking on a treadmill. Participants initially had twenty minutes of supine rest. Then, the ACBs were wrapped and set to 15 mmHg applied compression. According to our randomisation protocol, the ACB was either kept not actuated or was actuated for each test day. If the ACBs were actuated they applied higher compression. The participants stayed in the supine position (*baseline*) for seven more minutes. After twenty seven minutes of supine, participants were head-up tilted (*HUT*) to 70°, for ten minutes (*HUT1*). This was followed by walking on a treadmill at a speed of 26.8 m/min (1 mph) for five minutes (*walk*). Then, participants stepped back on the tilt-table and were head-upright tilted for a further ten minutes (*HUT2*). Finally, they were tilted back to the supine position for five minutes of recovery. The test was terminated if either: their systolic blood pressure fell below 80 mmHg; their HR was less than 50 bpm or greater than 180 bpm; they experienced presyncopal symptoms such as light-headedness, nausea, perspiration and warmth; or the entire protocol was completed. At test termination, the tilt table was rapidly returned to the supine position [18].

### Instrumentation

#### Active compression brace

The prototyped ACB is shown in Fig 1. Smart material technology, specifically SMAs, were utilized in the ACB design as the actuator. The SMA wires that were selected were 102 μm diameter low temperature Flexinol actuator wires (Dynalloy, Inc. Irvine, California, USA). The fabricated ACB also included a Boa closure system with a steel lace. The Boa system (Boa Technology Inc. Denver, Colorado, USA), shown in Fig 1, was placed at the two ends of the brace to fasten the ACB, which had negligible strain when tensioned. The ACB consisted of a thin layer of Teflon on top of a protective layer of Neoprene (forming the body of the brace) with SMA wires as the actuators. The 3D printed guides and fixtures held the SMA wires in place. The wires were fixed at the end of the brace by small washers and a conductive plate for electrical connection. The detailed conceptual design of the ACB is described in our previous works [23,29]

As an electrical current was applied to the SMA wires of the ACB, their module of elasticity increased and started to reduce in length–this in turn exerted a pressure on the calf via the Teflon sheet. Pressure exertion from the ACB consisted of two components: mechanical pressure and actuation pressure. The *mechanical pressure* was the initial pressure produced by wrapping the ACB around the leg firmly and fastening the Boa lace, which provided initial stress to the SMA wires. The *actuation pressure* resulted from the SMA wires when a current was applied to them. The total pressure exerted by the ACB on the calf was determined by summing the two components–mechanical pressure and actuation pressure–together.

Two PicoPress (MediGroup Australia Pty Ltd, Melbourne, Australia) pressure sensors were used to continuously measure the pressure exerted on both legs by the ACBs. A thin circular probe was attached to the pressure sensor. The probes were placed between the ACB and the calves of the participant at the center of the ACB, which was positioned over the maximal circumference of the calves.

The experimental setup included a driver circuit, which was coupled to a data acquisition system (DAQ) and a developed LabVIEW software, to pass the required current through to the ACB and receive the measurement signal. All signals were recorded using a NI 9205 analog input module (National Instruments, Austin, Texas, USA). The LabVIEW code performed multiple functions: it measured and recorded the applied current of the ACB, it determined the required voltage to be applied based on the test protocol at specific timing during the tests, it recorded the beat-to-beat arterial pressure signals, and it measured and recorded the calibrated strain-gauges plethysmography data.

Using this experimental approach, we were able to continuously target, and measure, the mechanical and actuation pressures. The pressure exerted by the ACB on the underlying PicoPress sensor was visualized using the PicoPress program. The ACB was then adjusted through the Boa system until the pressure while the limb was in a relaxed stationary supine position was consistent at 15mmHg. This pressure was considered to be the applied mechanical pressure, and would be subject to change in different phases during the muscle movement. For tests where the ACB was actuated, the actuation pressure was regulated by applying an electrical current of 3.6±0.08 A (180±4 mA per SMA wires), achieved by altering the output voltage to the ACBs using the DAQ and the LabVIEW program through the driver circuits. This applied current was the same for all participants, and was kept constant throughout the test. This means that the actuation pressure might be different for different shapes and compliances of the participants’ legs, and might change in different phases of the test (for example due to alterations in the heat loss rate for the SMA wires). It should also be noted that the actuation pressure is mainly result of an increase in the elastic modulus of the SMA wires due to the martensite to austenite phase transformation. For these reasons, we measured mechanical and actuation pressures throughout testing for each participant and present these data as group means.

#### Hemodynamic monitoring

Throughout testing we continuously recorded non-invasive beat-to-beat finger arterial pressure (Portapres, Finapres Medical Systems, Amsterdam, the Netherlands), which is based on photoplethysmography. The finger cuff was placed on the middle phalanx of the middle finger on the left hand. Alterations in finger pressure imposed by changes in the position of the hand with respect to heart level were compensated using the in-built height correction system. Continuous internal calibration was also conducted using the in-built Physiocal algorithm. Changes in CO and SV were also calculated from the recorded finger arterial pressure with the Beatscope software (Finapres) using the Modelflow technique [30]. This has been validated for evaluation of *changes* relative to baseline measurements [31], but without calibration against established techniques such as thermodilution, *absolute* values are not comparable [32]. Thus, we only report changes in these variables.

HR and rhythm were monitored using a lead II ECG; (Finapres ECG Module, Finapres Medical Systems, Amsterdam, the Netherlands).

For our primary outcome measures of SV and SAP we also quantified the orthostatic burden during the two tilted portions, as the area under the curve (AUC) of the response during the entire phase (excluding the first 25 and last 35 seconds of each phase where data may be less stable). AUC were calculated as the difference between the baseline parameter (the average of the first 20 seconds of the final 35 seconds before the end of baseline phase) and the values for each second of the test multiplied by the time.

We also determined limb volume changes using strain-gauge plethysmography; a simple and non-invasive method. Two strain gauges (D.E. Hokanson Inc., Bellevue, USA) were placed above the ACB around the left and right calves. Measurements of the circumference of the calves were conducted continuously throughout testing.

A standard tape measure and skinfold callipers (Slim GuideH, Creative Health Products, Plymouth, USA) were used to measure the largest circumference of the calf and skinfold thickness of the calf at its widest point. Measures were taken in triplicate on both legs, and the average was used for analysis.

### Data analysis

Data are presented as the average of the first 20 seconds of the final 35 seconds before the end of each test phase. For the ACB applied pressure, the average of the applied pressure on the left and right legs are reported. Data from three participants (one female, two males) were excluded from our analyses: one experienced presyncope during the protocol; one was unable to tolerate ACB activation at the desired pressure; and in one the ACB was a poor fit to their calves so desired pressures were not obtained. Strain gauge data (but not haemodynamic responses) were excluded for one additional participant due to technical difficulties. Data from one participant during HUT1 was excluded from the AUC calculation of SAP and SV since the measurement was interrupted during the first few minutes of the phase.

### Statistical analyses

All data were initially processed in MATLAB 2016Ra (The Mathworks Inc.) then transferred to JMP 12.2.0 (Statistical Analysis Systems Institute Inc.) for statistical analyses. Data were tested for normality using the Kolmogorov and Smirnov assumption and parametric or non-parametric testing used accordingly. Data are reported as means and standard error of mean. Significance was considered where p<0.05. Comparisons for the main effects of condition (ACB actuated or wrapped) and test phase (baseline, HUT 1, walk and HUT2) were conducted using repeated measures of ANOVA, with the Tukey post hoc test. Interaction effects were considered when the main effect was statistically significant. Multiple regression analyses were used to develop a predictive model for efficacy of the ACB, change in SV and HR, from selected anthropometric characteristics and ACB condition (wrapped or actuated). Two linear regressions were conducted for the initial sudden change, and the delayed gradual change of the calf circumference in HUT1. The intersection point was used to calculate separate contribution of distension of capacitance vessels (pooling) and capillary filtration [17] in the volume change of the calf.

### Evaluation of ACB comfort and wearability

Wearability of a device can be affected in several ways, and should be taken into consideration for the assessment of the device. These effects include physiological, comfort and biomechanical factors [33] [33]. In this study, we used a questionnaire-based method to evaluate comfort and wearability. Physiological effects were evaluated by participant self-assessment of the perceived exertion of the device. Comfort was evaluated using the comfort rating scales (CRS) proposed by Knight and Baber [34]. The CRS is explained briefly later in this section. Biomechanical effects determine if a wearable device predisposes the wearer to excessive musculoskeletal or postural load [33]. In this study, as the ACB was designed to apply load on the body, we only evaluated the compression exerted by the device on the calf as a marker of musculoskeletal loading; biomedical effects were excluded from our evaluation.

#### Descriptors of comfort

Several dimensions of comfort were considered encompassing six domains: four domains of physical perceptions (domains 2, 3, 4 and 5), and two domains of psychological perceptions (domains 1 and 6) of comfort. Domain-2 refers to nonharmful sensations of the device on the body; domain-3 refers to the harm caused by the device conveyed through sensations of pain; domain-4 refers to nonharmful feelings associated with the perception of being awkward, where the wearer makes conscious compensations or modifications to movements or actions; domain-5 refers to a direct impact of the device on normal movement patterns; domain-1 refers to psychological perceptions of discomfort; domain-6 refers to concerns related to the safety of wearing the device and its performance [34]. Table 1 shows the questions related to the six comfort domains that were used in the questionnaires.

**Table 1:** Questions for the CRSs evaluation [36].

Domain	Question Code	Description
(1) Emotions	E-1	I was feeling worried about how I look when I wore the ACB. I felt tense because I was wearing the ACB.
(2) Attachment	A-1	I was not able to move as usual.
	A-2	I had difficulty in putting on/off the device.
(3) Harm	H-1	The attached ACB caused me some kind of harm.
(4) Perceived change	Pc-1	Wearing the ACB made me feel physically different. I felt bulky.
(5) Movement	M-1	The ACB affected the way I moved. The ACB inhibited or restricted my movement.
(6) Anxiety	An-1	I did not feel secure with the ACB.
	An-2	I felt that I did not have the ACB properly attached.
	An-3	I felt that the ACB was not working properly.

Each domain was assessed using a 21-point scale, called CRS, anchored at each end with the labels “low” and “high”. A 21-point scale was used so that scoring on the scale would range from 0 at the far left to 20 at the far right [34].” According to Knight and Baber this range was considered large enough to elicit a range of responses that could be used for detailed analysis and fulfilled the criterion of [35], which recommended the use of scales with 11 or more points for subjective evaluation. In rating perceptions of comfort, each participant expressed his/her level of agreement, from low to high, with the statements made in the “description” column of Table 1. These statements were devised based on the interpretation of the aspect of comfort each within each domain.

#### Criteria for wearability

We evaluated the wearability of the ACB based on the criteria defined in Table 2. The level of effect ranging from low to extreme can be defined based on the value recorded from the evaluation. Borg CR-10 and CRS scales were used for the descriptors and the level of effect was categorised based on the value obtained, ranging from “low” to “extreme”. Five wearability levels [33] were used as shown in Table 3.

**Table 2:** Level of effects and corresponding wearability levels [36].

	Metric	Units	Level of Effect				
			Low	Moderate	Large	Very Large	Extreme
**Energy cost**	Relative perceived exertion	Borg CR-10 score	0–1	2–3	4–5	6–7	8–10
**Comfort**	General wearability	CRS score	0–4	5–8	9–12	13–16	17–20

**Table 3:** Level of effects and corresponding wearability levels [33].

Level of Effect	Wearability Level	Outcome
**Low**	WL1	System is wearable
**Moderate**	WL2	System is wearable, but changes may be necessary, further investigation is needed
**Large**	WL3	System is wearable, but changes are advised. Uncomfortable
**Very Large**	WL4	System is not wearable, fatiguing, very uncomfortable
**Extreme**	WPL5	System is not wearable, extremely stressful and potentially harmful

## Results

### Anthropometric measures

A summary of participant anthropometric measures and age is presented in Table 4.

**Table 4:** Mean and standard error of mean for anthropometric variables.

Age (y)	Height (cm)	Weight (kg)	BMI (kg/m^2^)	Right Calf Circumference (cm)	Left Calf Circumference (cm)	Muscle Cross Sectional Area (cm^2^)	Skinfold Right Calf Thickness (mm)	Skinfold Left Calf Thickness (mm)
27.9±1.3	172±2	68.6±1.9	23.2±0.4	37.4±0.4	37.2±0.5	83.1±3.8	11.8±1.4	11.9±1.3

### ACB pressures applied

**Fig 3:**
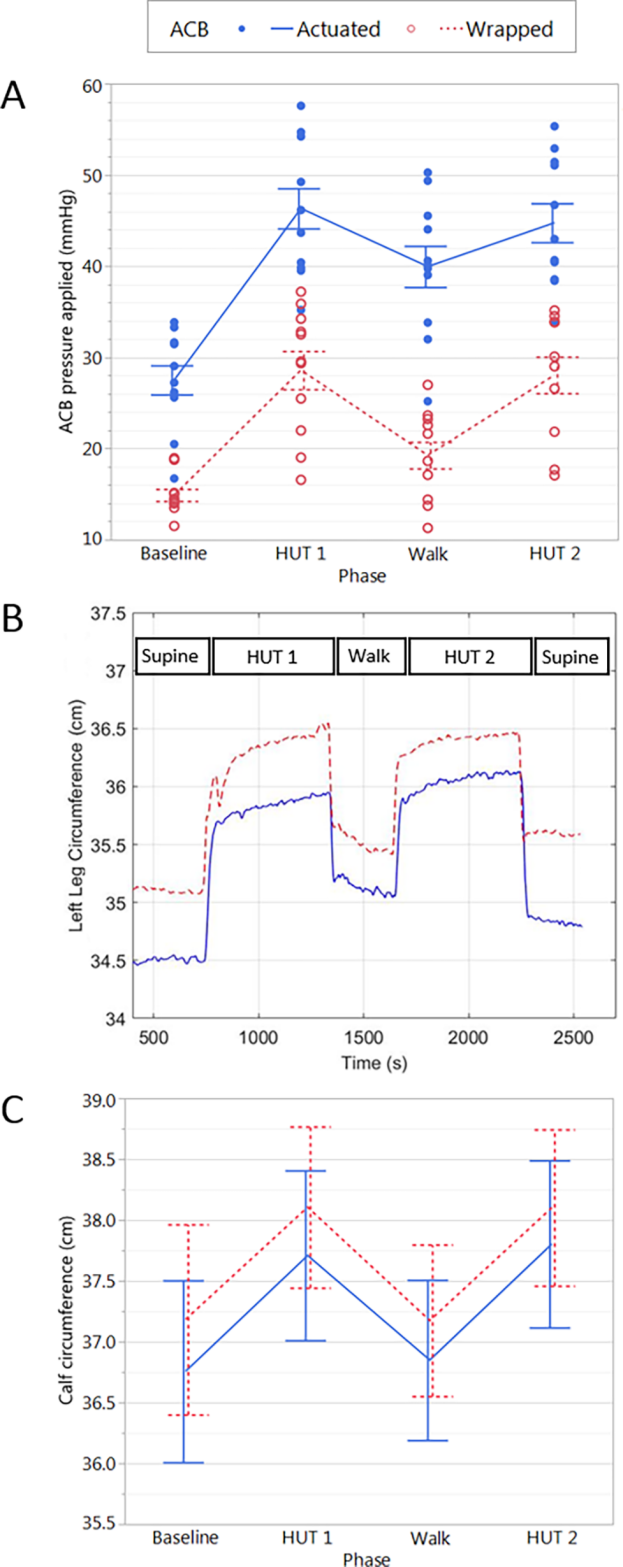
ACB performance and its impact on calf circumference changes. There was a significant main effect of ACB activation on the calf pressures obtained difference (p<0.001) (A). A representative example trace from one participant showing the impact of the ACB in wrapped and actuated conditions (B). Group data (left and right legs combined) showing the impact on calf circumference in the two experimental conditions are shown in (C). There was no significant difference in main effect of test actuation condition on calf circumference.

The ACB mean applied pressure, wrapped and actuated, for the four test phases are presented in Fig 3A. The mean actuation pressures (the increase in the applied pressure due to ACB actuation compared to the wrapped condition) were +12.6±1.5, +17.7±1.5, +20.7±1.7, and +16.7±1.3 mmHg during the baseline, HUT1, walk, and HUT2 phases of the experiments respectively (Fig 3A).

### Calf volume changes

A representative example trace from one participant showing calf circumference changes throughout testing can be seen in Fig 3B. In this individual, the circumference decreased -0.6±0.0 cm during the baseline phase when the ACBs were actuated. Group data are shown in Fig 3C. For the group as a whole there was no significant difference in the calf circumference between actuated and wrapped conditions. When we considered changes in calf circumference due to venous pooling (initial rapid increase in circumference) and capillary filtration (secondary slow increase in circumference) separately, there were no significant main effects of actuation on the responses obtained.

### Cardiovascular responses

**Fig 4:**
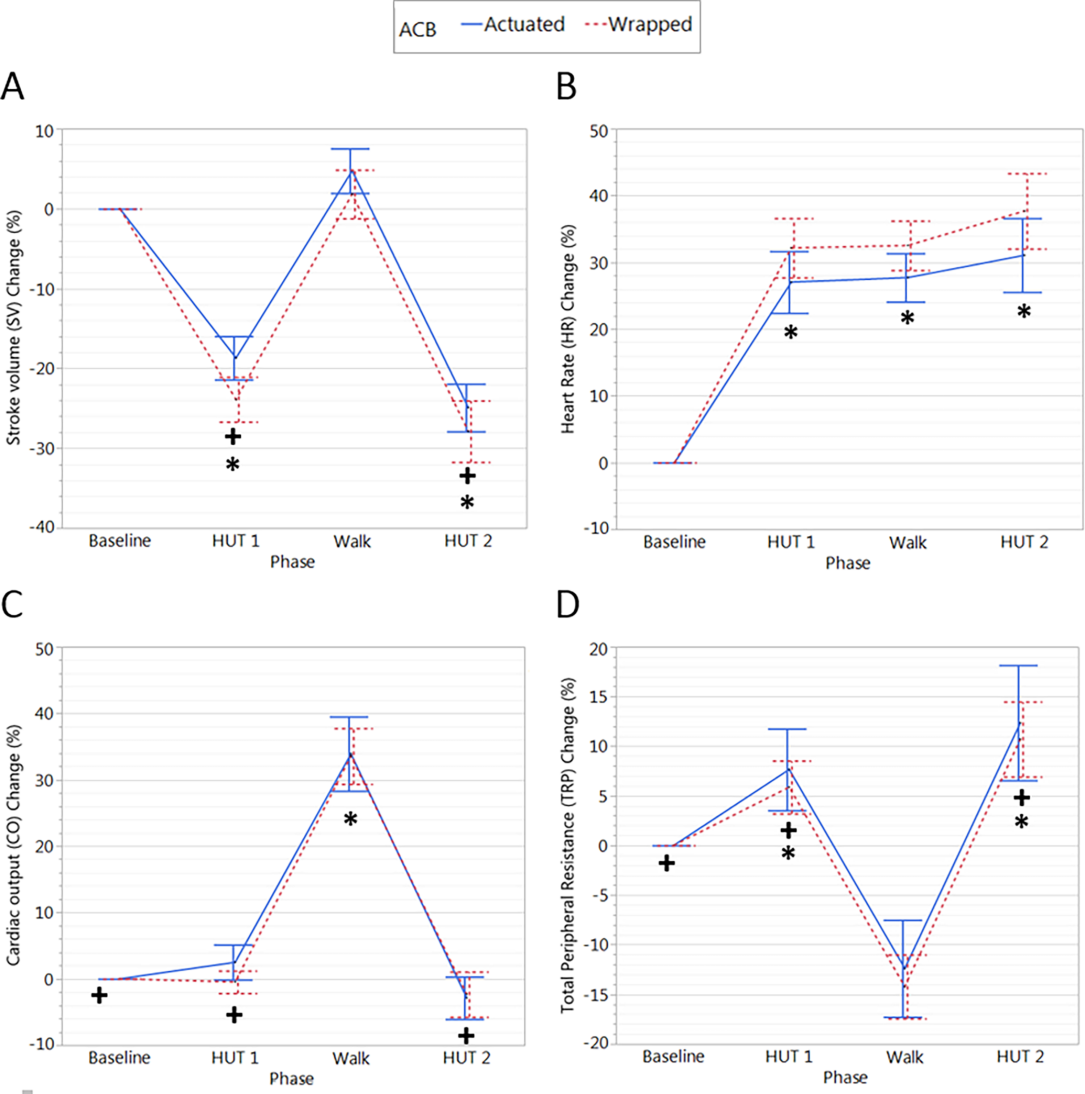
SV, HR, CO and TPR changes in the two test conditions. There was a significant main effect of test condition on HR change (%) (p = 0.038) and borderline significant main effect of test condition on SV change (%) (p = 0.061) during HUT1, walk and HUT2. Main effects of test phase are indicated: * denotes significant difference from baseline (p<0.05); + denotes significant difference from walk (p<0.05).

The impact of the actuation and wrapped conditions on cardiovascular responses during the test phases can be seen in Table 5. In addition, the changes in SV, HR, CO, and TPR compared to the baseline phase are illustrated in Fig 4.

**Table 5:** Cardiovascular responses during baseline, HUT1 and HUT2 in the two ACB conditions.

Parameter		Units	ACB	Phase				
				Baseline	HUT1		HUT2	
	ΔCO	l/min	Wrapped	-		-0.02 ± 0.11		-0.15 ± 0.23
			Actuated	-		0.13 ± 0.18		-0.27 ± 0.23
*	ΔSV	ml	Wrapped	-		-25 ± 3	×	-29 ± 4
			Actuated	-		-18 ± 2		-24 ± 2
	SAP	mmHg	Wrapped	111 ± 4		112 ± 4		110 ± 6
			Actuated	116 ± 5		123 ± 6		113 ± 5
	DAP	mmHg	Wrapped	50 ± 2		55 ± 3		57 ± 4
			Actuated	52 ± 2		59 ± 2		58 ± 2
	MAP	mmHg	Wrapped	66 ± 2		70 ± 3		72 ± 4
			Actuated	69± 3		75 ± 2		73 ± 2
*	PP	mmHg	Wrapped	61 ± 2		58 ± 2	**+ ×**	53 ± 3
			Actuated	64 ± 4		65 ± 5		56 ± 5
*	HR	beats/min	Wrapped	64 ± 3	**+**	84 ± 3		88 ± 4
			Actuated	71 ± 4		89 ± 4		92 ± 5
	TPR	mmHg.s/l	Wrapped	0.622 ± 0.038		0.663 ± 0.048	**+**	0.698 ± 0.058
			Actuated	0.615 ± 0.036		0.657 ± 0.038		0.683 ± 0.042

Data for SV and CO are shown as change relative to baseline values for each condition respectively. When considering the main effect of ACB actuation/wrapped condition over the three phases, there were significant differences between actuated and wrapped conditions in ΔSV (p = 0.008), pulse pressure (p = 0.045), and HR (p = 0.007). When considering the main effect of test phases for data collapsed according to ACB condition, there were significant differences between baseline and HUT1 in HR (p<0.05), between baseline and HUT2 in pulse pressure and TPR (p<0.05), and between HUT1 and HUT2 in ΔSV and pulse pressure (p<0.05). Abbreviations: CO, cardiac output; SV, stroke volume; SAP, systolic arterial pressure; DAP, diastolic arterial pressure; MAP, mean arterial pressure; PP, pulse pressure; HR, heart rate; TPR, total peripheral resistance.

* denotes significant difference between actuated and wrapped conditions when considering the main effect of ACB over the three phases (p<0.05)

*+* indicates denotes significant difference from baseline (p<0.05)

*×* denotes significant difference between HUT1 and HUT2 (p<0.05).

#### The effect of ACB actuation

**Fig 5:**
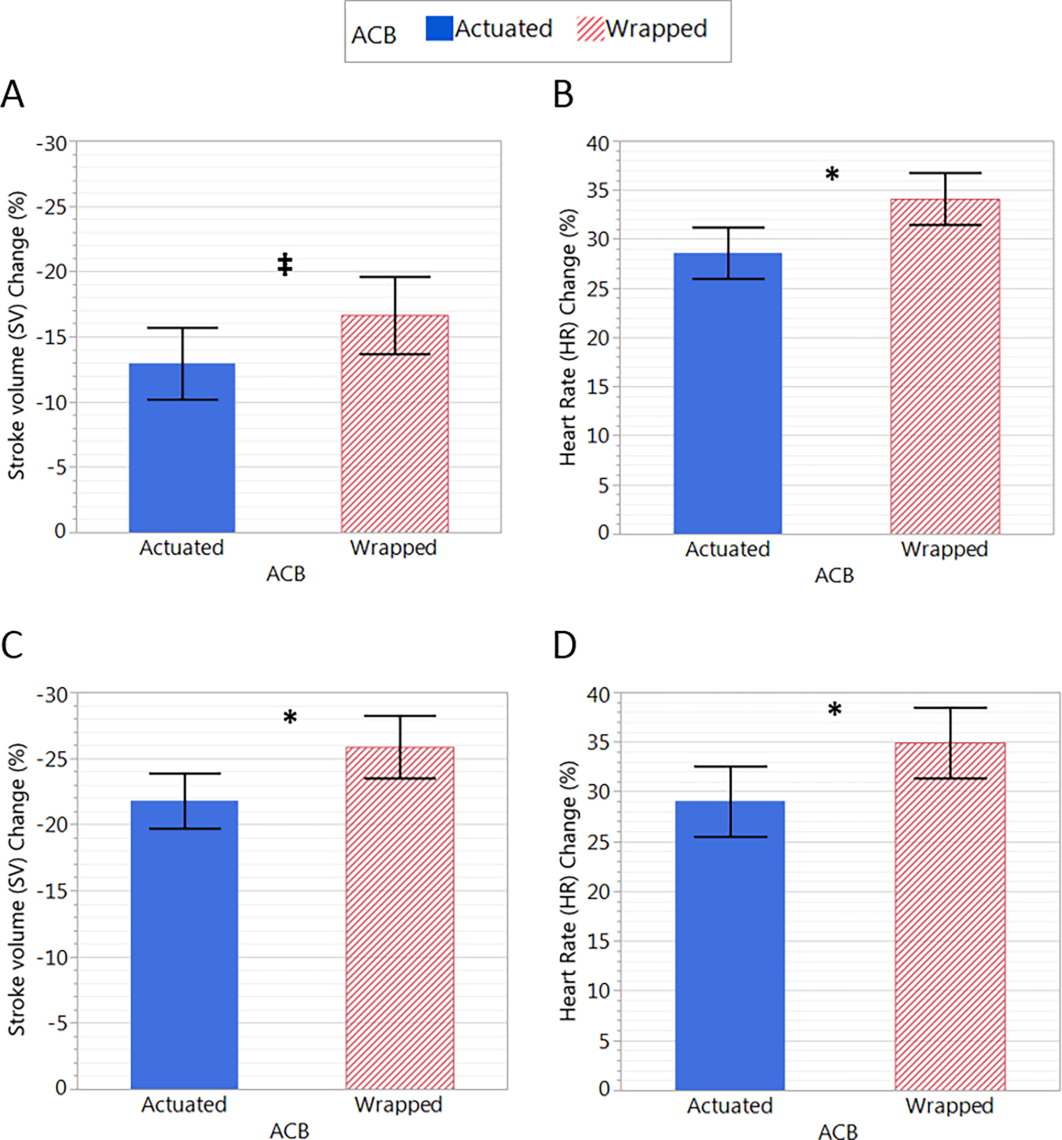
**Changes in SV and HR in the two conditions averaged over HUT1, walk and HUT2 combined (A & B) and averaged over the two HUT tests only (C & D).** There was a borderline significant main effect of test condition on SV change (p = 0.061) and significant main effect of test condition on HR change (p = 0.038) during HUT1, walk and HUT2 (A, B). There was a significant main effect of test condition on SV change (p = 0.037) and HR change (p = 0.038) when averaged during HUT1 and HUT2 only (C, D). Data are presented as the % change relative to baseline. * denotes a significant difference for the actuation condition (p<0.05); double dagger (‡) indicates a borderline significant difference (p = 0.061).

There were significant main effects of test condition, i.e. the difference between actuated and wrapped conditions, whereby the ΔSV (p = 0.008), and HR (p = 0.007) were reduced with ACB actuation, and the pulse pressure was larger with ACB actuation (p = 0.045) (Table 5). Because we were primarily interested in the impact of ACB actuation during orthostasis, we examined the average change relative to baseline in SV and HR during only the upright phases of HUT1, walk, and HUT2 (Fig 5A and 5B) as well as during only the tilted phases (HUT1 and HUT2) (Fig 5C and 5D). There was a significant main effect of test condition on HR change (p = 0.038) and borderline significant main effect of test condition on SV change (p = 0.061), whereby they were reduced with ACB actuation during HUT1, walk and HUT2 (Fig 5A and 5B). There was a significant main effect of test condition on both SV change (p = 0.037) and HR change (p = 0.038), whereby they were reduced during HUT1 and HUT2 (Fig 5C and 5D). When considering the differences between ACB actuation and placebo during tilt after supine rest there were trends for a larger stroke volume (+5.20±2.34%, p = 0.05) and lower heart rate (-5.12±2.41%, p = 0.06) with ACB actuation, with no effect on systolic arterial pressure (+4.86±3.41%, p = 0.18). There were no significant differences in any other cardiovascular parameters between test conditions.

**Fig 6:**
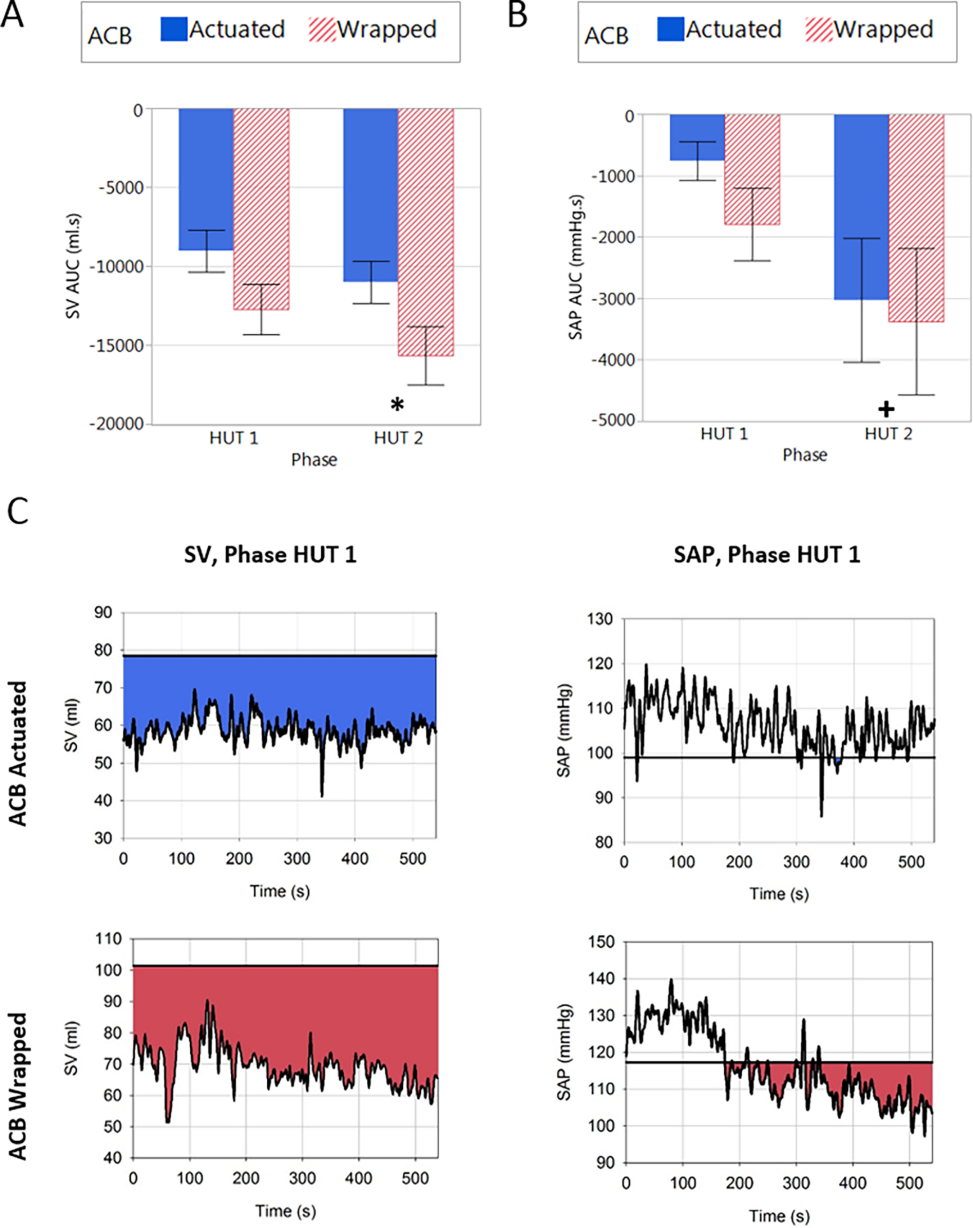
The cumulative area under the curve (AUC) during the different test phases in actuation and wrapped conditions. The cumulative area under the curve (AUC) can be seen during each test phase for SV (A) and SAP (B). There was a significant main effect of test condition (collapsed over the tilted phases) on SV AUC (p = 0.0011). Note the baseline shift in stroke volume in the wrapped condition–highlighting the need to evaluate stroke volume responses as changes relative to baseline, rather than absolute values, as described panels A and B, and in the manuscript. There was a significant main effect of test phase for SAP AUC, where the AUC was larger during HUT2 than HUT1 (p = 0.0161). Example traces from a representative individual for SV and SAP in the ACB wrapped and actuated conditions during phase HUT1 (C). Horizontal line indicates baseline SV and SAP for that individual and shaded area indicates regions below supine SV and SAP. * denotes a significant difference (main effect) between the actuation and wrapped conditions (p<0.05); + denotes significant difference (main effect) from HUT1 (p<0.05).

The cumulative AUC for SAP and SV are shown in Fig 6. There was a significant main effect of test condition on SV AUC (p = 0.0011), where the SV AUC was smaller in the actuated condition than the wrapped condition. When considering the interaction, the SV AUC was significantly smaller in the actuated condition than the wrapped condition in HUT2 (p<0.05). There was no effect of test condition on the SAP AUC.

#### The effect of phase of testing

Significant main effects between test phases, i.e. the differences between baseline, HUT1, HUT2 and walking phases, can be seen in Fig 4. During the walking phase, CO was significantly increased compared to baseline (ACB wrapped: +33.5±4.2%; ACB actuated: +33.9±5.6%). TPR was significantly reduced in the walk phase compared to baseline (ACB wrapped: -14.2±3.2%; ACB actuated: -12.4±4.9%). HR was increased during walking compared to baseline (ACB wrapped: +32.5±3.7%; ACB actuated: +27.7±3.6%). SV was not different during walking compared to baseline.

There was no significant effect of the two tilting phases on CO compared to baseline. SV was significantly decreased during both HUT1 and HUT2 compared to baseline (ACB wrapped: -23.9±2.8 and -27.9±3.8%; ACB actuated: -18.7±2.7 and -24.9±3.0% in HUT1 and HUT2 respectively). HR was significantly increased during HUT1 and HUT2 compared to baseline (ACB wrapped: +32.2±4.4 and +37.7±5.6%; ACB actuated: +27.0±4.6 and +31.1±5.5% in HUT1 and HUT2 respectively. TPR increased significantly compared to baseline in both HUT1 and HUT2 (ACB wrapped: +5.9±2.7 and +10.7±3.8%; ACB actuated: +7.6±4.1 and +12.4±5.8%). When considering the cumulative AUC for SAP and SV (Fig 6.) there was a significant main effect of test phase only for SAP AUC, where the AUC was larger (p = 0.016) during HUT2 than HUT1.

#### Relationships between SV, HR change (%) and anthropometric variables

**Fig 7:**
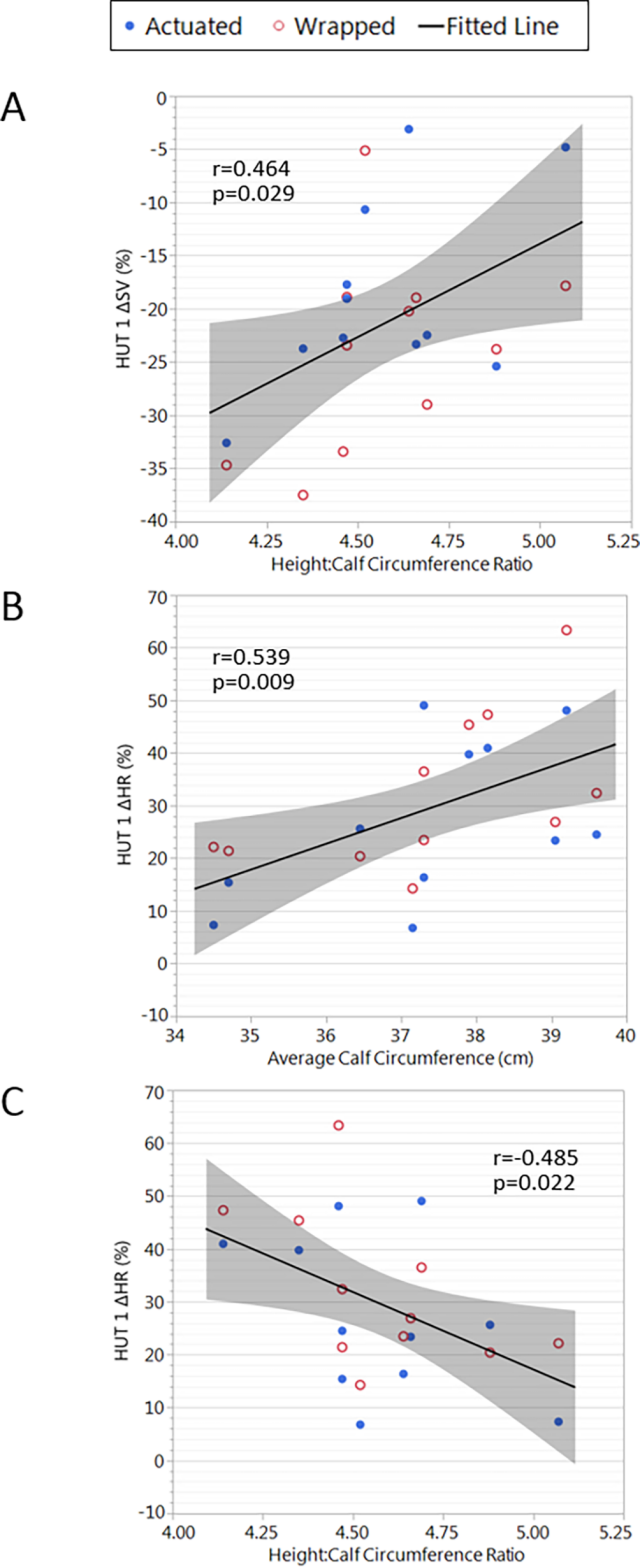
Relationships between SV, HR change (%) and anthropometric variables. Data are presented as the % change relative to baseline.

Considerable variability was noted between individual responses for change in SV (%) and HR (%). We evaluated whether this was related to anthropometric variables. During HUT1, the change in SV (%) was positively correlated with the height:calf circumference ratio (Fig 7A). The change in HR (%) was positively correlated with the average calf circumference and was negatively correlated with the height:calf circumference ratio (Fig 7B and 7C).

There was no significant relationship between the change in SV during HUT1 and the calf circumference measurements when expressed as average calf circumference, muscle cross-sectional area and height:muscle cross sectional area ratio. Similarly, there was no significant relationship between the change in HR during HUT1 and the calf circumference measurements when expressed as muscle cross-sectional areas and height:muscle cross sectional area ratio. However, there was a significant relationship between the change in HR (%) and height:calf circumference ratio (p = 0.022, R = -0.485); further analysis showed that average calf circumference was negatively correlated to the height:calf circumference ratio (p = 0.015, R = -0.512). These relationships between anthropometric characteristics and haemodynamic control were no longer significant when considered after the walking phase.

### The ACB comfort and wearability evaluation

#### Physiological

**Fig 8:**
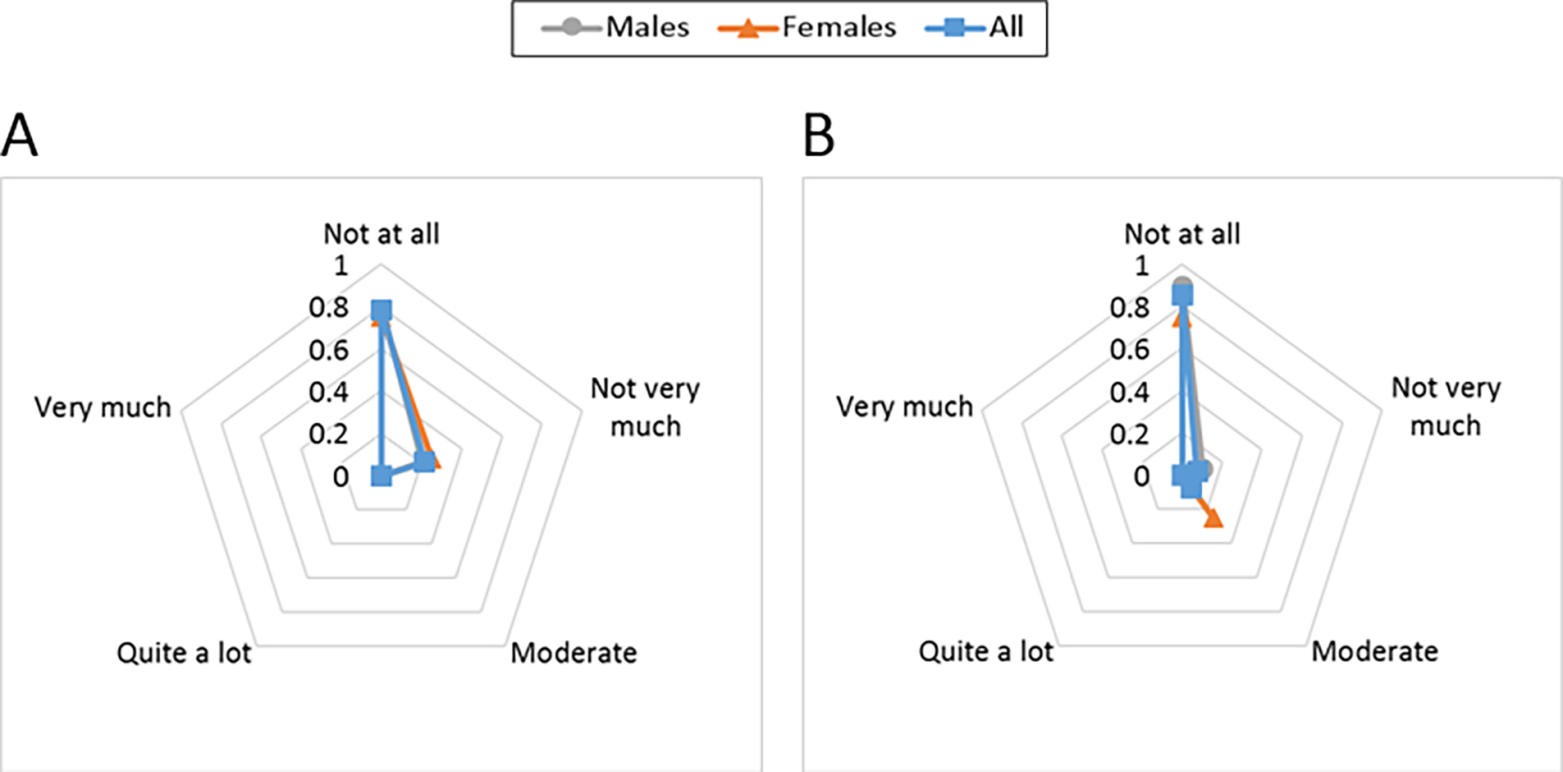
The ACB wearability evaluation. Results for the perceived exertion evaluation with the ACB actuated (A) and the ACB wrapped (B).

Most participants did not feel the ACB was causing “exertion” in either wrapped or actuated conditions (Fig 8). When the ACB was actuated, one female and two male participants expressed physical stress. When the ACB was wrapped, two participants expressed feelings of extra exertion.

#### Comfort

**Fig 9:**
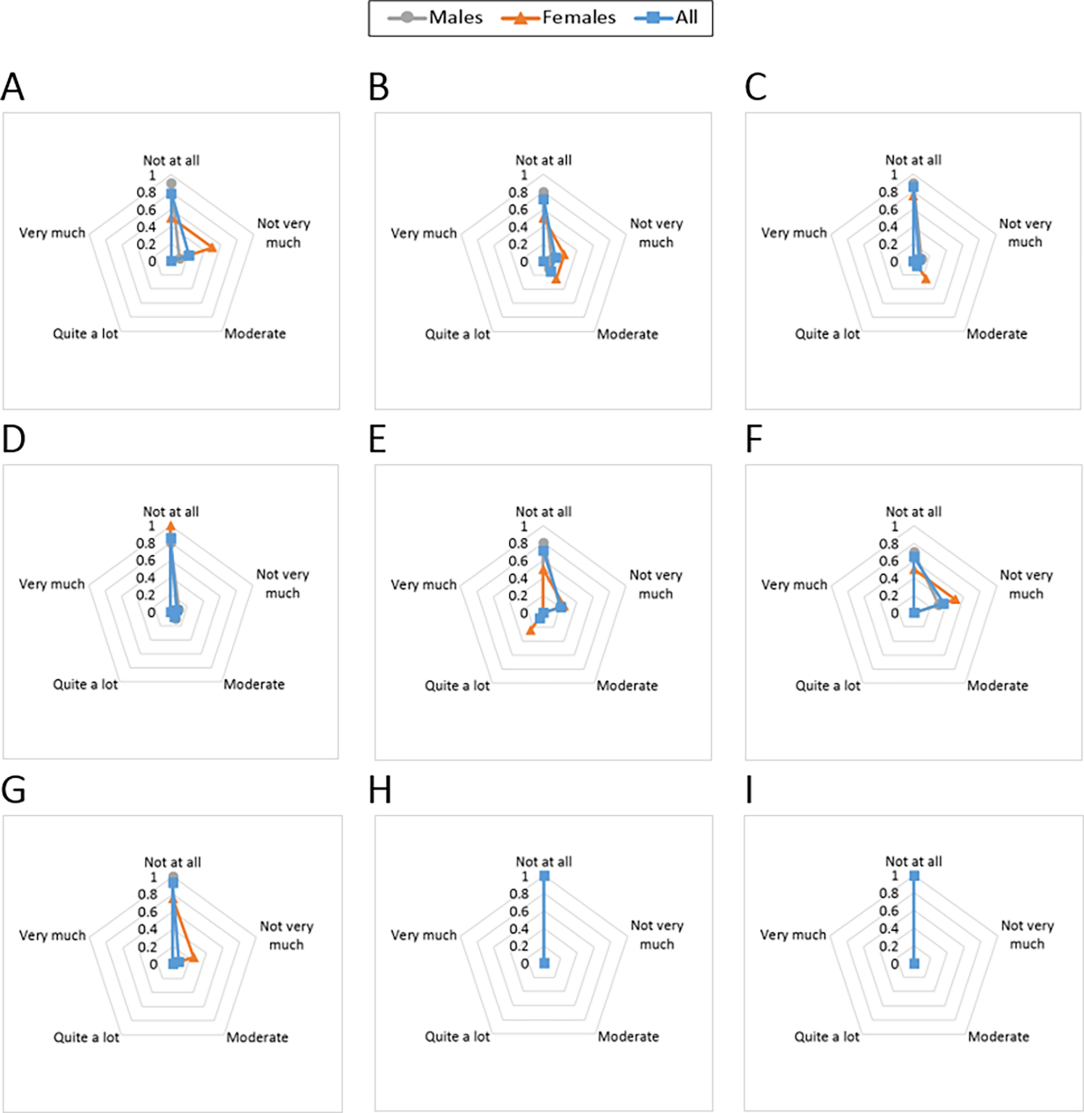
The ACB comfort evaluation. Comfort results are shown for each of the domains of emotion, attachment, harm, perceived change, movement and anxiety. The participants were asked to rate their agreement with each of the following statements: “I was feeling worried about how I look when I wore the ACB. I felt tense because I was wearing the ACB.” (A); “I was not able to move as usual.” (B); “I had difficulty in putting on/off the device.” (C); “The attached ACB caused me some kind of harm.” (D); “Wearing the ACB made me feel physically different. I felt bulky.” (E); “The ACB affected the way I moved. The ACB inhibited or restricted my movement.” (F); “I did not feel secure with the ACB.” (G); “I felt that I did not have the ACB properly attached.” (H); and “I felt that the ACB was not working properly.” (I).

Results from the comfort evaluation can be seen in Fig 9.

#### Emotion

Fig 9A presents the participants responses to the questions related to emotion. Most of the participants did not feel tense or worried about how they looked wearing the ACB. Two of the females and one male participant had a moderate emotional concern.

#### Attachment

Most participants were unconcerned by the attachment of the ACB to their body (Fig 9B and 9C). In terms of movement, two participants (one male and one female) felt the device stopped them from moving as usual and two participants (one male and one female) reported they felt moderately uncomfortable. This feeling includes the sensation of having the device on their bodies and its movement. Regarding the comfort in wearing the device, only one female participant felt it was difficult to put on/off and one male participant was moderately uncomfortable. Results show that attachment scored relatively higher for the female participants compared to the males.

#### Harm

Results regarding any kind of harm such as pain, heat, itching, and irritation, caused by the ACB are illustrated in Fig 9D. None of the female participants expressed any feeling of being harmed, but there were two males who reported moderate to high concerns of harm.

Perceived change: The feedback from the participants on the perceived change is presented in Fig 9E. One female participant expressed that the device felt “quite a lot” bulky. Also, there were four other participants with moderate concern that the device felt bulky, two males and two females.

#### Movement

The concerns of the participants in terms of movement restriction are reflected in Fig 9F. Five participants (three males) expressed experiencing moderate obstruction of their movement when wearing the ACB. The rest of the participants did not show any concerns regarding this aspect.

#### Anxiety

Answers to the questions related to anxiety arising because of wearing the ACB are shown in Fig 9G,9H and 9I. None of the participants expressed any anxiety that the ACB was not properly attached or was not working properly. One female participant felt moderately insecure wearing the ACB.

#### Overall evaluation

The overall level of effect of the ACB on participants is summarized in Table 6. It is observed that on average all the domains had a low level of effect. Target areas for future improvement are perceived change and attachment, and to a lesser extent, harm and movement.

**Table 6:** Overall comfort and wearability evaluation results.

	Metric	Unit	Score	Level of Effect
**Energy cost**	Perceived Exertion (ACB Actuated)	Borg CR-10 score	0.68 ± 0.26	Low
	Perceived Exertion (ACB Wrapped)	Borg CR-10 score	0.64 ± 0.3	Low
**Comfort**	Emotion	CRS score	1.71 ± 0.74	Low
	Attachment	CRS score	3.11 ± 0.63	Low
	Harm	CRS score	2.36 ± 0.82	Low
	Perceived change	CRS score	3.21 ± 1.00	Low
	Movement	CRS score	2.79 ± 0.76	Low
	Anxiety	CRS score	0.57 ± 0.17	Low

## Discussion

We have demonstrated that the prototyped ACB was effective at improving SV in a randomised, double-blind, placebo-controlled study on healthy participants. The test protocol was designed to investigate the efficacy of the ACB during passive head-up tilting, in which blood pooling occurs, and walking, in which the skeletal muscle pump increases venous return.

### ACB pressures applied

The ACB can apply a desired wrapping compression similar to commercial compression stockings, 15 mmHg, and when altering the compression by actuation, can apply pressure up to 27.3±1.6 mmHg, in the supine resting position. In both HUT 1 and 2 phases applied pressures showed a significant increase compared to the baseline phase (p<0.05). This could be because of the muscle movements, contraction, and an increase in calf circumference. The maximum applied pressure was observed in HUT1 and HUT2 phases when the actuated ACB applied pressure of 46.3±2.2 and 44.8±2.1 mmHg respectively. The walk phase showed lower applied pressure as a result of a drop in calf circumference. This decrease could be because of an increase in venous return due to the muscle pump. It should be mentioned that walking also altered the performance of the ACB due to increased heat loss in the SMA wires. Walking increases the convective heat transfer coefficient because of the air flow, which decreases the degree of actuation (phase transformation) in the SMA wires. In fact, this alteration could potentially be useful as a self-regulatory factor of the ACB since during walking, the muscle pump mechanism is active and there might be less of a need for compression.

### Calf volume changes

In both HUT1 and HUT2 phases there was an initial rapid and secondary gradual increase in calf circumference that is thought to be due to venous pooling and capillary filtration respectively [17]. In the walking phase, it was observed that the calf circumference decreased due to muscle contractions and enhanced venous return. There were no significant differences in either calf volume changes, or changes attributed to venous pooling or capillary filtration, between the actuated and wrapped conditions.

### Cardiovascular responses

CO was maintained throughout each test, except during walking, when it increased markedly. The maintenance of CO in the HUT1 and HUT2 phases likely reflects the intact baroreflex response in these healthy control volunteers, whereby reductions in SV were accompanied by compensatory increases in HR and vascular resistance. Indeed, the increases in HR during the tilted phases closely matched the decreases in SV. This is compatible with a reduced venous return when upright, secondary to venous pooling and plasma filtration [18], and a consequence decrease in SV.

There were significant main effects of test condition on changes in SV and HR during tilting, whereby the SV was lower and HR higher in the wrapped than the actuated conditions. This effect was less clear when the walking phase was incorporated, presumably because in this case SV volume was mainly maintained because of the muscle pump mechanism and the impact of device actuation was reduced. These data highlight the need for compression garment paradigms that can be applied selectively, only during passive orthostasis, and not during active orthostatic manoeuvres such as walking when any deficits in cardiovascular reflex control may be offset by the action of the skeletal muscle pumps [37].

There was no significant effect of the actuation condition on blood pressure responses or orthostatic burden (SAP AUC). This also likely reflects the intact baroreflex response in healthy volunteers, in whom blood pressure is well maintained during brief mild orthostatic stresses such as those encountered in the present study. However, the higher associated stroke volume and lower heart rates during orthostatic stress in the actuation condition suggest that this maintenance of blood pressure was achieved with a smaller baroreflex response, compatible with the actuation condition augmenting stroke volume, and presumably, venous return.

When considering the possible relationships between SV, HR changes and anthropometric variables we first examined interactions between the main conditions (ACB wrapped/actuated) and each variable during initial passive orthostatic stress. Since we found no differences in the slopes of these relationships between the two test conditions, we combined data for the two ACB conditions. We showed that individuals with larger calf circumferences had higher heart rate responses to orthostatic stress, associated with larger reductions in SV. Therefore, is tempting to speculate that those with larger calf circumferences would be poised to benefit most from ACB actuation; however, because there were no differences in the slopes between the regressions for actuated and wrapped conditions, this is not the case. The relationships between haemodynamic parameters and anthropometric variables were no longer significant during the second tilt phase, which was preceded by walking. Presumably there is an effect of walking and associated autonomic and muscle activation on either orthostatic haemodynamic control or calf circumference that persists for some time (at least ten minutes) after termination of the activity. This has implications for the use of the device during activities of daily living–device actuation may not be necessary during walking because the enhanced skeletal muscle pumping and autonomic activity seem sufficient to maintain cardiovascular control. We suggest that actuation of the device upon cessation of activity would be of more benefit.

### The ACB comfort and wearability

Overall the ACB comfort and wearability evaluation showed a low level of concern in all the domains considered. Although overall the device was well tolerated, some participants expressed discomfort, and priority areas for improvement in this regard were identified (attachment and perceived change, and to a lesser extent, harm and movement).

### Limitations

Our physiological tests were designed to be double-blind in the sense that the primary investigator was unaware of the test condition during data collection and analysis, and the participants were not told what to expect from the compression conditions. However, although participants were not informed whether the ACB was actuated or not on each test day, nor were they told the expected outcome of the experiment, it is possible that the study was not truly double-blinded. The ACB gets a bit warm when it is actuated, and participants may have been aware of a different sensation due to the higher compression.

It is possible that we have underestimated the impact of ACB actuation, because we compared it to a wrapped condition, which also applied a low level of pressure, and may itself have reduced orthostatic fluid shifts and enhanced orthostatic cardiovascular control. Comparisons of ACB actuation with no pressure application might show even greater benefit. However, we wished to compare whether our device provided additional benefit over and above conventional strategies that employ a low level of calf compression similar to our wrapped condition. In addition, use of a wrapped condition had the benefit that for participants and experimenters the protocol was the same for both tests, enhancing our ability to blind the study. Additional considerations for future study might also include the incorporation of a true placebo condition in which no compression was applied.

We tested healthy volunteers, and we can not be certain if the results would extend similarly to a patient population. For example, in healthy controls pooling and filtration is lower during orthostatic stress than in patient populations [17], and so the potential to improve cardiovascular control by ameliorating these effects might be expected to be reduced. However, we believe our results may extend to patients with orthostatic intolerance, because a few of our control participants actually showed high calf volume changes more comparable to those inpatient populations. Furthermore, other non-pharmacological approaches to prevent or delay syncope apply equally well to both patients and controls [38,39].

We had a relatively small sample size in this preliminary study proof-of-concept study, with an obvious impact on statistical power. As a consequence, we only considered the main effects of the test condition and phase, and not the interaction effects. Future studies should further elucidate these interactions.

It may be that the application of compression stockings prior to rising in the morning would have a greater effect, due to the ‘‘water jacket effect”, whereby oedema accumulating during the day restricts further venous pooling [40]. However, it has been shown that 20 minutes of supine rest is sufficient to normalise any prior venous pooling/capillary filtration effect [17], at least in control participants, so we consider this unlikely. The possibility for distal oedema below the compression level should also be considered in future work.

We evaluated the efficacy of the ACB on improvement of SV and calf volume changes, and accordingly our results may not extend to other compression garments. The existing literature suggests that compression garments extending to the thigh and abdomen may be more effective at preventing orthostatic intolerance [8,11,13,14], but are associated with poor patient satisfaction and compliance [15,16]. Future studies may wish to examine the optimum compromise between efficacy, comfort, and patient compliance.

Our limited sample size in this preliminary evaluation precluded the evaluation of potential sex differences in terms of blood pooling and cardiovascular response to ACB actuation. Comparison between male and female participants’ responses and the efficacy of ACB should be considered in future, particularly given the known differences in venous pooling and capillary filtration between men and women [41] and increased susceptibility to orthostatic intolerance in women [42].

## Conclusions

This paper showed the feasibility of an ACB to achieve the desired external calf pressure, and demonstrated an enhancement of stroke volume and reduction in orthostatic tachycardia during device actuation. In addition, evidence was provided on wearability, comfort, viability and ambulatory use of the ACB in human participants.
